# Ganglion cyst at the proximal tibiofibular joint - A rare cause of compression neuropathy of the peroneal nerve

**DOI:** 10.1016/j.radcr.2021.10.004

**Published:** 2021-11-02

**Authors:** Ghazn Khan, Zeeshan Kazmi, Bushra Khan, Nadir Khan, Shalini Datta

**Affiliations:** a Manchester Medical School, Faculty of Biology, Medicine and Health, Stopford Building, Oxford Road, University of Manchester, Manchester, UK; b Macclesfield District General Hospital, East Cheshire NHS Trust, Macclesfield, UK; c Royal Alexandra Hospital, Department of Radiology, NHS Greater Glasgow and Clyde, Paisley, UK

**Keywords:** Ganglion cyst, tibio-fibular joint, cystic lesion, common peroneal nerve, nerve compression

## Abstract

Ganglion cysts are fluid filled sacs which develop near joints and tendons and are usually asymptomatic. Lower limb ganglion cysts are rare occurrences especially those situated around joint spaces causing nerve compression. We present the case of a 68 year-old female with history of progressive swelling in the left antero-lateral leg, associated with pain, and neurological symptoms of peroneal nerve compression. Magnetic resonance imaging (MRI) revealed a large proximal tibiofibular joint ganglion cyst causing peroneal nerve compression. One year following the left sided presentation, the patient presented with similar but less severe symptoms in her right antero-lateral leg. MRI revealed a small juxta-articular ganglion cyst in the right proximal tibiofibular joint space. We discuss etiology, symptoms, and management of lower limb ganglion cysts.

## Introduction

Ganglion cysts are fluid filled structures which develop near joints and tendons. They are usually asymptomatic and rarely require any treatment [1]. Neuronal compression can be particularly troublesome resulting in palsies, paresthesia or loss of function depending on the degree of compression and the particular nerve affected [2]. The proximal tibiofibular joint is a rare site for ganglion cyst development and thus, compression of the peroneal nerve due to a ganglion cyst is a poorly documented occurrence [3,4]. Prompt recognition and treatment are essential in managing this condition and its complications. In this paper, we report a patient with a ganglion cyst as a rare cause of peroneal nerve palsy and discuss its pathology, imaging findings and management.

## Case report

A 68-year-old female presented to the orthopedic outpatient clinic with a several-month insidious onset of a lump over the lateral aspect of her left knee, with associated lateral based knee pain symptoms. These were initially severe however subsided into a more chronic state with associated nocturnal issues. Additionally, the patient began to experience localized sensory symptoms over the dorsal aspect of the left 3rd, 4th and 5th toes, with sparing of the web spaces and the rest of the dorsum foot. She also reported being prone to tripping after prolonged walking as well as a mild foot drop. The patient has no prior history of knee surgery or trauma, and the only past medical history of note is long term hypertension, for which she has been managed with antihypertensive medication.

On clinical examination, the patient had full range of motion in her knee. There were no signs of joint effusion and the knee was stable on both collateral and cruciate ligament testing. There was some mild medial joint line tenderness but no lateral joint line tenderness. She had a large palpable mass over the proximal lateral aspect of her lower leg as well as pain around the proximal tibiofibular joint. Lower limb neurological examination revealed 4 of 5 power in her ankle dorsiflexion and 5 of 5 power in her great toe dorsiflexion, using the Medical Research Council's scale (MRC scale) of muscle power [5].

Magnetic resonance imaging (MRI) of the left knee without contrast was performed using a protocol of T1-weighted and Proton Density Fat Suppressed weighted images, in the sagittal, coronal, and axial planes.

**Fig. 1:**
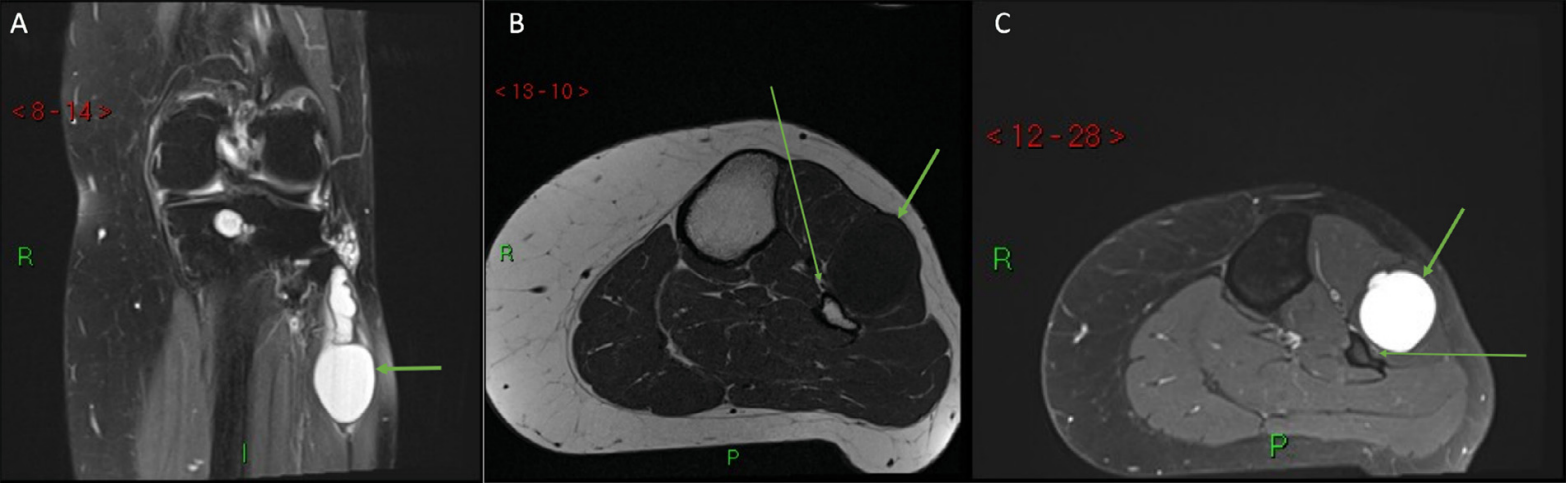
(A, B, C): Left knee MRI in coronal Proton Density Fat Suppressed (A), axial T1-weighted (B) and axial Proton Density Fat Suppressed (C) showing a multi-loculated ganglion cyst anterolateral to the proximal fibula (thick arrow) causing peroneal nerve compression (thin arrow). R is right; I is inferior; P is posterior.

Images revealed a large multi-loculated ganglion cyst anterolateral to the proximal fibula, measuring 3 cm (anteroposterior) by 2.8 cm (transverse) by 7 cm (cranio-caudal), causing peroneal nerve compression (Fig. 1). Additionally, there were background degenerative changes with medial osteophytosis, narrowing of the medial compartment joint space and a large subchondral cyst present within the posterior aspect of the medial tibial condyle. There was also a small incidental tear through the posterior horn of the medial meniscus.

Nerve conduction studies were done using electromyography (EMG) on the left leg which displayed present but reduced left common peroneal and left sural sensory responses. In addition, left common peroneal motor response to extensor digitorum brevis was absent when stimulated at the ankle, fibula head and popliteal fossa. No voluntary motor units could be recruited in the left extensor digitorum brevis.

Following the EMG studies and MRI, the results were discussed with the patient, and it was agreed that the patient would not undergo surgical excision. The patient decided this based on the associated risks of surgery ie, infection, pain, ongoing disability, common peroneal palsy and permanent nerve damage. She was recommended for conservative management with regular follow-up at the outpatient clinic.

A year following the left-sided presentation, the patient presented with new symptoms of pain over the antero-lateral aspect of her right leg and paresthesia in the lateral border of the right foot. On examination, there was a mild degree of swelling in the anterolateral aspect of her right proximal tibia, with altered sensation in the lateral three toes but normal motor power. The symptoms in her left leg remained unchanged with slight increase in size of swelling.

**Fig. 2:**
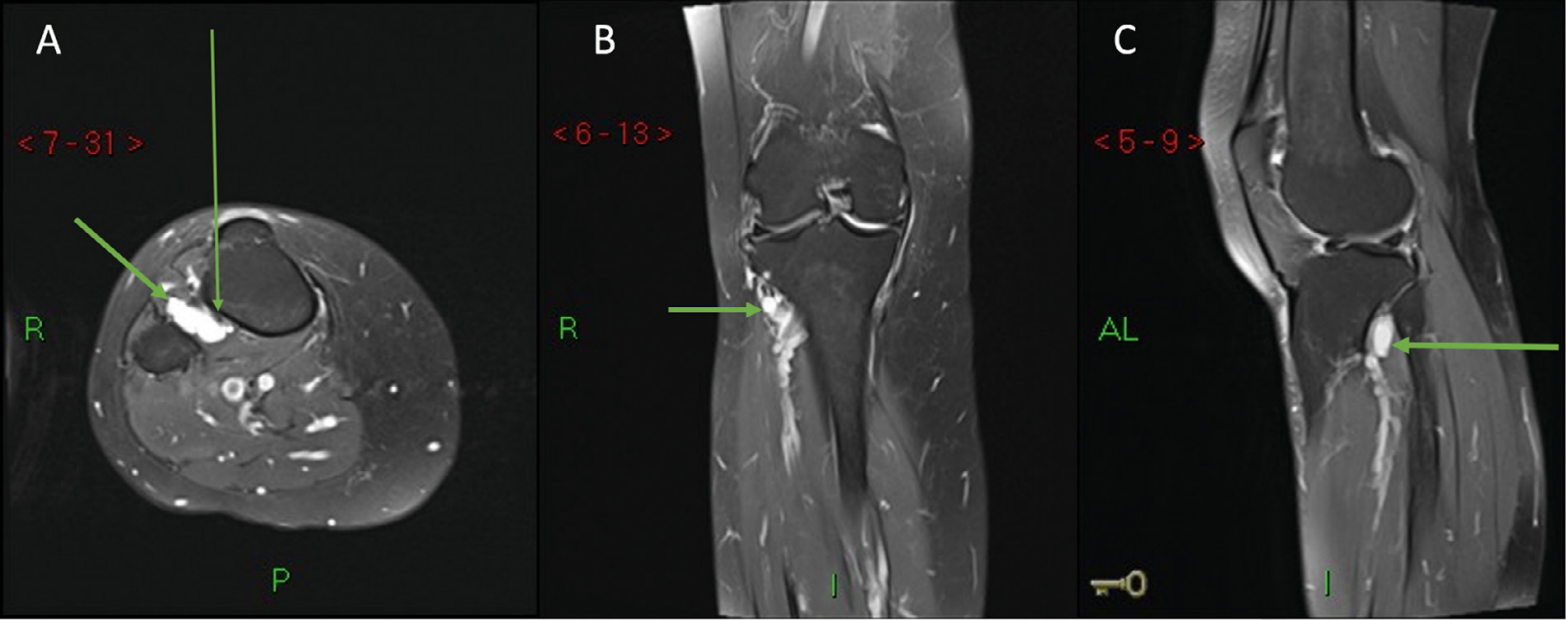
(A, B, C): Right knee MRI in axial (A), coronal (B) and sagittal (C) Proton Density Fat Suppressed showing a juxta-articular ganglion cyst (thick arrow) located in the interosseus space immediately inferior and in communication with proximal tibiofibular joint space. The cyst is causing compression of the deep peroneal nerve (thin arrow). R is right; AL is antero-lateral; P is posterior.

MRI without contrast of the right knee was completed, using a protocol of T1-weighted in sagittal plane and Proton Density Fat Suppressed weighted images in the sagittal, coronal and axial planes. This revealed a well-defined lesion measuring 1.1 cm (anteroposterior) by 1.7 cm (transverse) by 1.8 cm (craniocaudal) in the interosseous space immediately inferior and in communication with proximal tibiofibular joint space (Fig. 2). The appearance was in keeping with a juxta-articular ganglion cyst. The image further showed compression of the anterior tibial vein and the deep peroneal nerve.

The patient was conservatively managed with pain relief medication for neuropathic pain and is being followed up in clinic. To this date no surgical excision or decompression has been performed in either leg.

## Discussion

The proximal tibiofibular joint, also known as the superior tibiofibular joint, is a plane type joint which consists of the intersection between the head of the fibula and the lateral condyle of the tibia. There is some gliding motion able to take place at this joint however, the main function of the joint is to assist in stability of the lower limb while weight bearing [6,7]. The joint is contained within a joint capsule alongside three ligaments: anterior superior tibiofibular ligament, posterior superior tibiofibular ligament and lateral collateral ligament of the knee. Furthermore, neurovascular structures are present near the joint. These include the common peroneal nerve and the popliteal artery which may be affected in cases of joint pathology and/or injury [7,8]. The most common pathology of the proximal tibiofibular joint is joint instability characterized by either subluxation or dislocation [9]. Other less common pathologies include ligamentous diseases, muscle weakness and peroneal nerve compression [7].

Ganglion cysts are non-malignant, fluid-filled sacs that commonly present in the hand and wrist [1,10]. Those originating from the proximal tibiofibular joint are much rarer with an estimated prevalence of just 0.76% [3].

The cause of ganglion cysts remains unclear, but is believed to be a result of degenerative changes within the connective tissue associated with joint capsules and tendon sheaths [1]. Histologically they appear as translucent masses, with a thin connective tissue capsule filled with mucinous material, that give rise to focal areas of chronic inflammation [11]. A number of risk factors have been identified that include female gender and repeated traumatic exposure [12,13]. They present as smooth lumps under the skin and are often painless. However, in some cases they can cause pain and discomfort.

Involvement of the common peroneal nerve due to compression leading to neuropathy is a rare event with very few cases reported in the literature [4,[14], [15], [16], [17]]. One case report described a 50-year-old woman with progressive foot drop and swelling over the fibular head which was confirmed to be ganglion cyst on imaging. This produced a peroneal nerve palsy which was relieved following surgical removal of the cyst [16]. A second review focused on two cases of peroneal nerve neuropathy due to compression via ganglion cysts. The two patients had similar findings of unilateral muscle weakness leading to loss of ankle dorsiflexion. Both patients had surgical excision of their respective cysts however, only one of the patients made a full recovery with the other continuing to have symptoms 18 months after surgery [15]. Similarly, a case report of a 50-year-old male with acute foot drop reported a compressive ganglion cyst affecting the peroneal nerve [4].

MRI is the preferred diagnostic test for ganglion cysts. It provides accurate localization and delineation of the lesion and distribution of the muscles supplying the peroneal nerve, due to the excellent soft tissue contrast. This is important for surgical planning. Lesions appear as unilocular or multilocular, lobular fluid collections and present with low signal intensity on T1-weighted sequences and high signal intensity on T2-weighted sequences [11]. In cases of common peroneal nerve palsy, denervated muscle demonstrates hyperintense T2 signal [2]. Nerve conduction studies using EMG are a helpful adjunct to imaging tests as it allows clinical findings to be correlated, by eliciting the extent of sensory and motor impairment that may be present.

Ganglion cysts do not usually require treatment, and many will resolve on their own. For painful cysts, aspiration or surgery may be recommended [1,18]. Surgery generally involves excision of the ganglia and the mass in its entirety. This is not without risk. The most common adverse effect associated with surgical resection is local recurrence. This can be avoided through articular branch ligation [14]. Other rarer complications include perineural fibrosis, traction injuries and in the most severe cases, complete nerve transection resulting in loss of function [14]. This is likely due to proximity of the peroneal nerve to the origin of ganglion cysts within the proximal tibiofibular joint.

## Conclusion

While ganglion cysts arising in the upper limb are common and well documented in the literature, a lower limb ganglionic cyst arising in the tibiofibular joint space is a rare finding that should be noted on routine imaging, especially when causing symptomatic peroneal nerve compression. These can be debilitating, and surgical excision is the preferred treatment. This however carries the risk of nerve damage, and risk of recurrence.

## Patient consent

Written, informed consent for publication of the case was obtained from the patient.
